# Insertion of intra-oral electrodes for cranial nerve monitoring using a Crowe–Davis retractor

**DOI:** 10.1007/s10877-016-9904-y

**Published:** 2016-07-05

**Authors:** Terrence L. Trentman, Christopher Thunberg, Andrew Gorlin, Antoun Koht, Richard S. Zimmerman, Bernard Bendok

**Affiliations:** 1 Department of Anesthesiology, Mayo Clinic Arizona, 5777 E Mayo Blvd, Phoenix, AZ 85054 USA; 2 Department of Anesthesiology and Neurological Surgery, Northwestern University Feinberg School of Medicine, Chicago, IL USA; 3 Department of Neurosurgery, Mayo Clinic Arizona, Phoenix, AZ USA

**Keywords:** Acoustic neuroma, Glossopharyngeal nerve, Hypoglossal nerve, Cranial nerve monitoring, Bulbar palsy

## Abstract

Acoustic neuroma resection is an example of a neurosurgical procedure where the brainstem and multiple cranial nerves are at risk for injury. Electrode placement for monitoring of the glossopharyngeal and hypoglossal nerves during acoustic neuroma resection can be challenging. The purpose of this report is to illustrate the use of a device for intra-oral electrode placement for intraoperative monitoring of the glossopharyngeal and hypoglossal nerves. A 60-year-old male presented for acoustic neuroma resection. Under general anesthesia, a Crowe–Davis retractor was used to open the mouth, providing access to the posterior pharynx. For glossopharyngeal monitoring, two bent subdermal needle electrodes were inserted just lateral to the uvula. Two additional electrodes were inserted on the lateral tongue to monitor the hypoglossal nerve. Cranial nerves monitoring was conducted utilizing both free running and triggered electromyography of the trigeminal and facial nerves in addition to the lower cranial nerves. The tumor was resected successfully. Monitoring of the cranial nerves (including the glossopharyngeal and hypoglossal nerves) revealed no concerning responses. The Crowe–Davis retractor and the technique described allowed insertion of electrodes for neural monitoring, contributing to neural preservation.

## Introduction

Assessing and maintaining the integrity of neural pathways is vital during neurosurgical procedures. Acoustic neuroma resection is an example of a neurosurgical procedure where the brainstem and multiple cranial nerves (CN) are at risk for injury. This includes not only the vestibulocochlear (CN VIII) and facial (CN VII) nerves [1], but in large tumors the lower cranial nerves as well, including glossopharyngeal, vagus, accessory and hypoglossal (CN IX–XII respectively).

Tumors of the 8th cranial nerve typically arise within the internal auditory canal, but can also arise within the cerebellopontine angle (CPA). Large tumors (>3 cm) are more likely to impinge on adjacent neural structures including the 5th cranial nerves and the lower cranial nerves as well as the brainstem. A substantial body of literature describes techniques and outcomes of intraoperative monitoring (IOM) of CN VII–VIII, which are most at risk during acoustic neuroma resection, with potential hearing loss and facial paralysis [2–8]. Monitoring of the glossopharyngeal and hypoglossal nerves, which has been infrequently described in the literature, requires intra-oral placement of recording electrodes. Injury of the lower cranial nerves is associated with bulbar palsy and related co-morbidities, including postoperative aspiration. A review of 333 patients undergoing vestibular schwannoma microsurgery found transient lower cranial nerve deficit in 6 % of the patients, versus 45 % of the patients who suffered facial nerve dysfunction immediately postoperatively (and 33 % at last follow-up) [9]. Other morbidities noted in this study were cerebral spinal fluid leak (63 %), headache (9 %) and epidural hematoma (3 %).

Although there is substantial risk associated with posterior fossa surgery, for large tumors including those that may cause compressive symptoms and even hydrocephalus, non-invasive treatments are less likely to be of benefit. The dose of radiation needed to treat a large acoustic neuroma may be unacceptably high. To reduce the risk of bulbar palsy from surgery, CN IX, X and XII monitoring can be employed. However, depending on patient anatomy, body habitus, the presence of an endotracheal tube, bite block and temperature probe, the insertion of intra-oral recording electrodes can be difficult and susceptible to dislodgement during the procedure. The purpose of this report is to illustrate the use of a device (the Crowe–Davis retractor) for intra-oral electrode placement for IOM of the glossopharyngeal and hypoglossal nerves. The patient provided written consent to publish this case report.

## Case description

A 60 year-old, 96 kg male (body mass index 27.8) with a 2 cm right acoustic neuroma with mass effect on the brainstem was scheduled to undergo tumor resection under general anesthesia. The patient had a 5-year history of progressive hearing loss and tinnitus in his right ear. He was taken to surgery where general endotracheal anesthesia was induced; routine vital sign monitors were used in addition to a radial arterial line. Anesthesia was maintained with <0.5 MAC of sevoflurane plus remifentanil and propofol infusions. No neuromuscular blocking agents were administered after intubation.

Cranial nerves monitoring was conducted utilizing both free running and triggered electromyography (EMG) of the trigeminal (masseter muscle) and facial nerves in addition to the lower cranial nerves. Cranial nerve monitoring for VII was accomplished from needles placed at the mentalis, orbicularis oris and oculi muscles. The nerve itself was stimulated intermittently throughout the surgery, including from the internal auditory canal to the brainstem, and on the tumor capsule. This was done because the tumor may splay the nerve and portions of it may be found in various places in or around the tumor. Monitoring of CN VIII was carried out with brainstem auditory evoked potentials (BAEP). To identify a positive (triggered) response after stimulation, a compound action potential is observed on the EMG waveform.

**Fig. 1:**
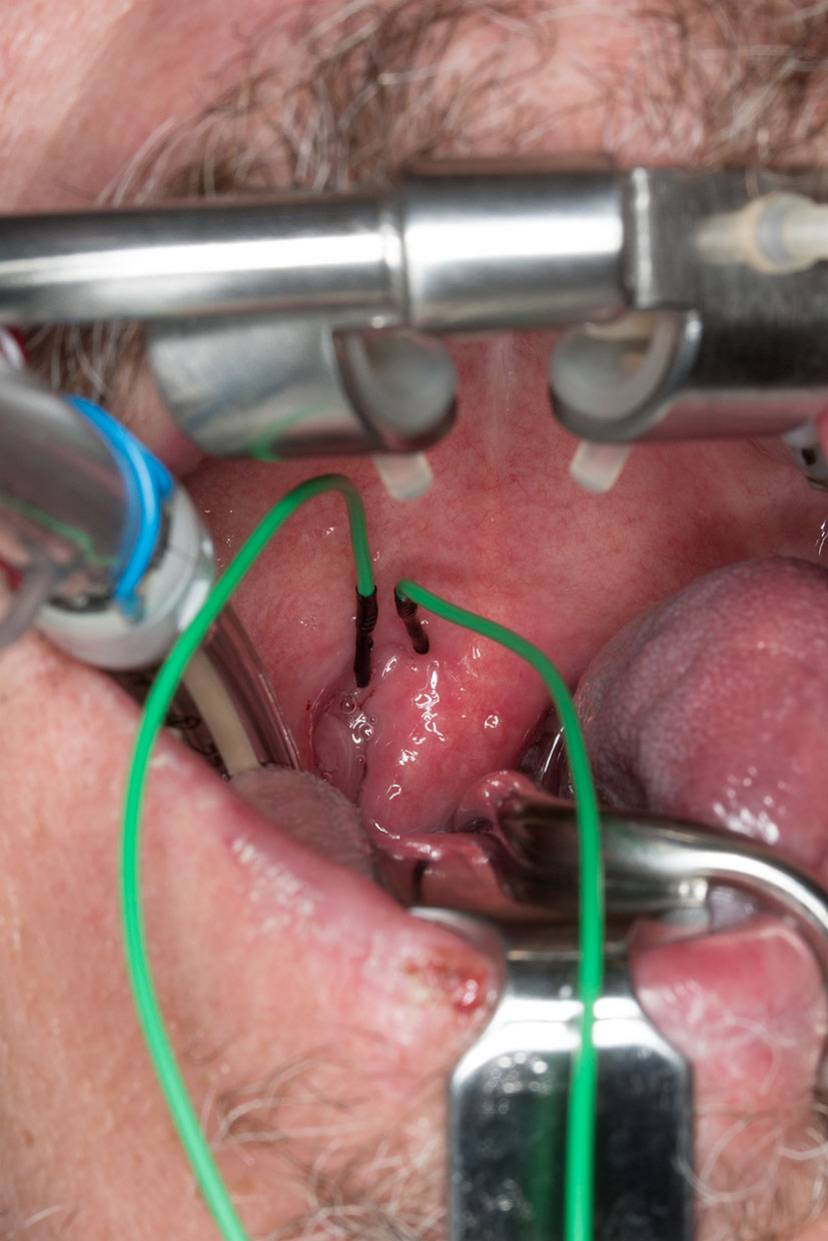
Cranial nerve IX monitoring electrodes inserted just lateral to the uvula, ipsilateral to the tumor. Electrodes are approximately 0.5 cm apart

**Fig. 2:**
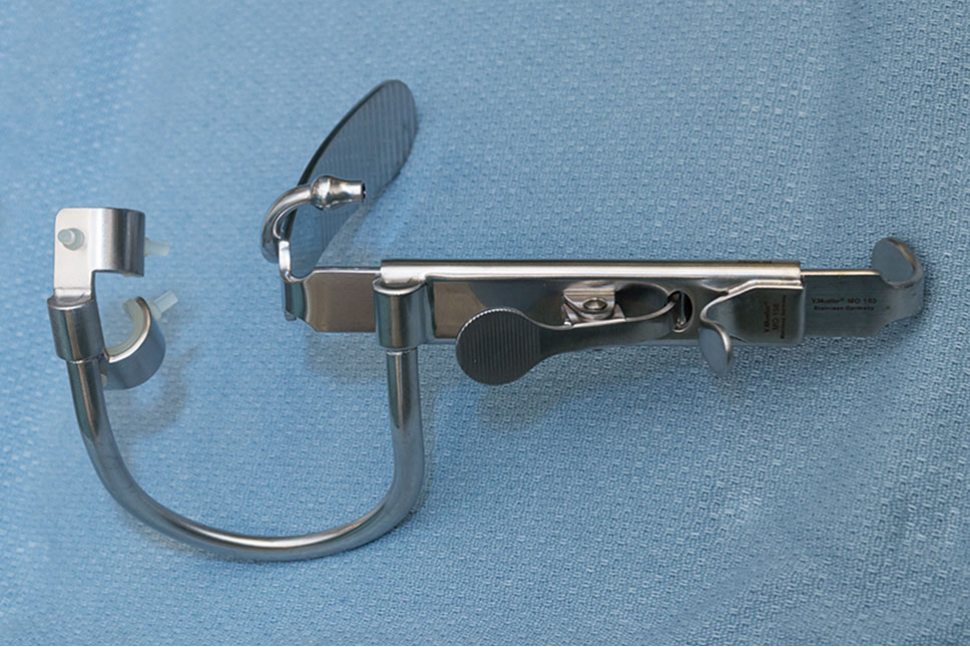
Crowe–Davis retractor (or “mouth gag”) with tongue blade, grooved jaws for the incisors and ratchet mechanism

**Fig. 3:**
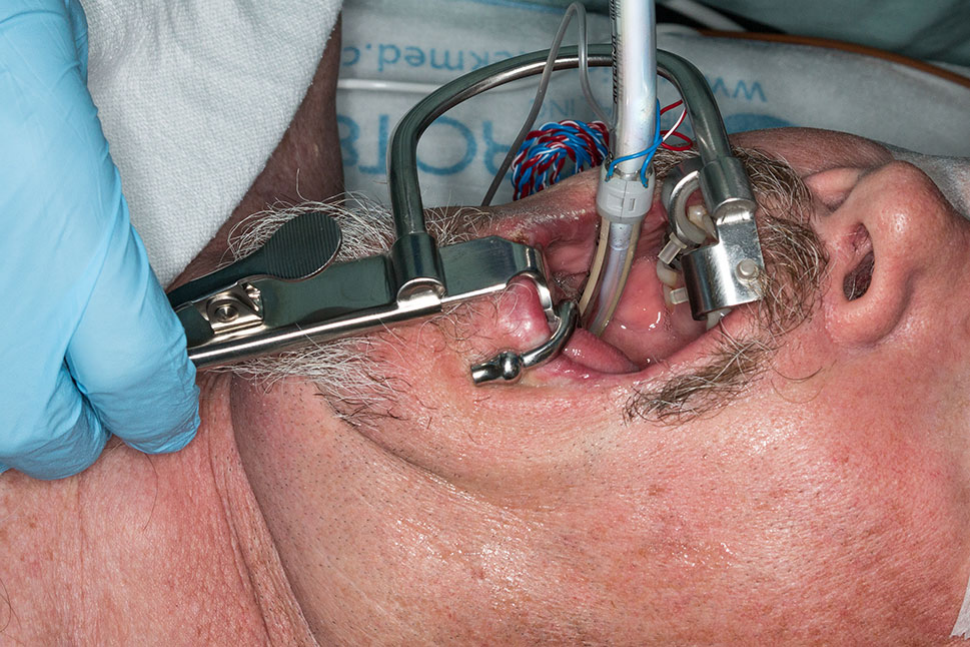
Mouth opened with Crowe–Davis retractor

Cranial nerve IX function was monitored with bent subdermal needle electrodes inserted approximately 0.5 cm apart in the soft palate, just lateral to the uvula and medial to the tonsillar pillar. This was done to monitor motor component of CN IX, the stylopharyngeus muscle (Fig. 1). A Crowe–Davis retractor was used to open the mouth widely after intubation to aid with insertion of the intra-oral electrodes, which in our practice is carried out by the anesthesiologist (Figs. 2, 3). The retractor has padded slots for the incisors and a blade to control the tongue. Various lengths of tongue blades are available. A ratchet mechanism allows slow and controlled mouth opening [10].

Bent subdermal needle electrodes (Rhythmlink, Columbia, SC, USA) were selected in an effort to minimize the risk of displacement, and tape is used on the face to further protect and stabilize the wires. A small clamp was used to grasp and insert the needle. As described elsewhere [11, 12], CN X integrity was monitored with a neural integrity monitor electromyogram endotracheal tube (NIM^®^ EMG, Medtronic, Minneapolis, MN, USA), which was placed under direct vision during routine laryngoscopy and taped to the face to prevent movement. Cranial nerve XI was monitored via recording electrodes placed in the trapezius muscle. Cranial nerve XII function was monitored with 2 bent subdermal needle electrodes inserted in the lateral aspect of the tongue, on the ipsilateral side. Both upper and lower extremity somatosensory evoked potentials (SSEP) were monitored during the procedure using the median, ulnar and posterior tibial nerves.

The procedure was carried out via a StealthStation^®^ (Medtronic, Minneapolis, MN, USA) guided right suboccipital approach. The intra-auditory canal was opened and CN VII was dissected completely off the tumor from both the brainstem and lateral approach. The neural integrity monitor (triggered EMG) demonstrated stimulation of CN VII at the level of 0.4 mA or greater as facial nerve anatomy was identified. While a much lower level of stimulation (e.g. 0.05 mA) has been reported during these cases and may indicate normal facial nerve function, in our case a higher threshold was needed. During the procedure, the monitoring technician typically alerts the surgeon of the need for higher thresholds to stimulate the facial nerve.  The surgeon can reassess the site of stimulation for proximity to the nerve, remove fluid from the field that may dissipate current, and evaluate function of the neurostimulator. Monitoring of the other cranial nerves (including CN IX and XII) revealed no concerning responses.

At the end of the procedure the electrodes were removed without difficulty, the patient was awakened, extubated and transported to the recovery room. The Crowe–Davis retractor was not needed to remove the bent subdermal needle electrodes, although this was a consideration if there was any difficulty. Immediately postoperatively, he was noted to have mild right-sided facial/labial weakness, which was felt to be due to surgical manipulation and edema. No evidence of dysphagia symptoms to liquids or solids was seen. He was discharged home on postoperative day 3 in good condition. At most recent follow-up (4 months postoperatively), he was noted to be doing well with near compete resolution of his facial nerve paresis.

## Discussion

This report illustrates monitoring of the lower cranial nerves during the resection of acoustic neuroma and other cerebellopontine angle tumors, most notably a device to aid insertion of intra-oral recording electrodes for CN IX and XII monitoring. While the frequency of lower cranial nerve monitoring is unknown, technical challenges are a disincentive to these techniques. The use of a Crowe–Davis retractor has made this process much easier for us, since the tongue, endotracheal tube and other soft tissue can make insertion of the needles challenging. This is particularly true of CN IX; the retractor allows access to the soft palate that would be difficult or impossible to achieve with other methods, especially in obese patients. The retractor can be used for safer electrode removal at the end of the procedure in certain patients as well, although removal does not require as much precision or perfect visualization.

Mishler and Smith mentioned but did not illustrate use of a Crowe–Davis retractor to facilitate electrode suturing for CN IX and XII monitoring [13]. They favored suturing the electrodes in the mouth with the aid of the retractor, although we have not found this to be necessary as we used bent subdermal needle electrodes.

The risk of false information from misplaced electrodes is what makes direct vision and the use of the Crowe–Davis retractor important. Dislodged needles will result in a silent EMG (f-EMG) as silence is the normal response (or possibly by changed impedance or noise); therefore, maintaining the needles is essential for proper monitoring. Securing the location of the needles utilizing the bent subdermal needle electrodes and placing them under direct vision will help maintain good monitoring, particularly since it can be very difficult to replace a dislodged needle during surgery.

Bulbar palsy (and postoperative respiratory complications) is a risk of acoustic neuroma resection, particularly in large tumors that require manipulation of the brain stem for complete resection. Duane et al. [14] reported 102 cases of acoustic neuroma, 9 of which were found to have postoperative bulbar palsy. Five of the patients suffered pulmonary complications including aspiration. They concluded that bulbar palsy was more likely to occur when the tumor was ≥3 cm. While lower cranial nerve monitoring is not currently a standard of care or carried out with the frequency of facial nerve monitoring, lower cranial nerve injury has a significant negative impact on patient quality of life.

Use of the Crowe–Davis retractor is not without risk. Laceration or contusion of the posterior pharyngeal wall is possible, as is temporomandibular joint (TMJ) dislocation. Monitoring cranial nerves is also associated with some risks, requiring a collaborative effort to ensure safety. Stimulation of CN IX at high thresholds can trigger reflex hypotension and bradycardia [13]. Cranial nerve XI can cause excessive motor responses of the trapezius and sternocleidomastoid muscles, resulting in head movement at inopportune moments [13]. Finally, as with any needle puncture, there is a risk of bleeding and hematoma formation.

In this case, use of a Crowe–Davis retractor allowed easy and secure insertion of oral monitoring electrodes for CNs IX and XII. We believe the future of neuromonitoring for large acoustic neuromas will include more routine CN IX, X and XII monitoring.
